# A Review of Flexible Wearable Antenna Sensors: Design, Fabrication Methods, and Applications

**DOI:** 10.3390/ma13173781

**Published:** 2020-08-27

**Authors:** Mariam El Gharbi, Raúl Fernández-García, Saida Ahyoud, Ignacio Gil

**Affiliations:** 1 Department of Electronic Engineering, Universitat Politècnica de Catalunya, 08222 Terrassa, Spain; mariam.el.gharbi2@upc.edu (M.E.G.); raul.fernandez-garcia@upc.edu (R.F.-G.); 2 Information Technology & Systems Modeling Team, Faculty of Sciences, Abdelmalek Essaadi University, 93002 Tetouan, Morocco; sahyoud@uae.ac.ma

**Keywords:** antenna sensor, textile materials, wearable antenna sensor, flexible

## Abstract

This review paper summarizes various approaches developed in the literature for antenna sensors with an emphasis on flexible solutions. The survey helps to recognize the limitations and advantages of this technology. Furthermore, it offers an overview of the main points for the development and design of flexible antenna sensors from the selection of the materials to the framing of the antenna including the different scenario applications. With regard to wearable antenna sensors deployment, a review of the textile materials that have been employed is also presented. Several examples related to human body applications of flexible antenna sensors such as the detection of NaCl and sugar solutions, blood and bodily variables such as temperature, strain, and finger postures are also presented. Future investigation directions and research challenges are proposed.

## 1. Introduction

The industrial and academic world have generated a lot of interest in the field of wearable and flexible electronics in recent years [1]. Flexible electronics, whose mechanical properties include to be wrinkled, bent, and stressed/collapsed, would considerably extend the applications of modern electronic devices to multiple real nonflat scenarios [2], including the shape of the human body [3]. As a consequence, flexible electronics combined with textile materials offer many advantages that make them an attractive technology for boosting the next generation of consumer electronics, among them are low-cost manufacturing, inexpensive flexible substrates, light weight, and ease of fabrication [4].

Recently, there has been a lot of interest focused on antenna sensors due to their simple configuration, multimodality sensing, passive operation, and low cost [5]. Antenna sensors are electronic devices with dual functionality for communicating and sensing, and they can be implemented by minimizing the number of components. The function principle of antenna sensors is demonstrated by their geometrical or intrinsic material change influence in terms of their antenna resonance frequency, which is evaluated by means of the impact on the reflection coefficient. In addition, antenna sensors have been evolved as another process to measure diverse physical parameters. To provide the wireless communication required by today’s information-oriented community, it is necessary to integrate flexible antenna sensors into flexible electronic systems [6].

In the literature, the first antenna sensor is published in [7]. A circular antenna is proposed to measure the humidity content of sludge samples. Above the antenna sensor, the sludge sample was placed inside a plastic beaker. The Bottcher model was used to calculate the humidity content of a sample from measurements of the dielectric effective permittivity of the antenna sensor. Several antenna sensors have been reported in the literature for measuring the relative permittivity of soils and snow [8], gas [9], relative humidity [10], pH [11], soil moisture [12], and glucose [13]. All of these antenna sensors can be categorized as antenna dielectric sensors. The relation between the physical measurement and the radiation parameters of the antenna is determined by the properties of the material. This issue cannot be modeled analytically in the majority of the cases [14], and it is one of the challenges of the development of antenna dielectric sensor. Therefore, almost all the reported works of antenna dielectric sensors are mainly based on direct characterization and experimental tests. With regard to antenna temperature sensors, the first one found in the literature is devoted to temperature threshold detection [15]. The antenna sensor was fabricated from a shape memory polymer (SMP) paper sandwiched between a radio frequency identification (RFID) tag and a metallic sheet. The shape memory polymer changed its relative permittivity as the temperature crossed a doorstep, which in turn could be recognized from the turn-on power of the RFID tag.

Up to now, antenna sensors are applied for many applications, such as agricultural activities and gardening, structural health, biomedical sensing, food quality monitoring, and so on, which are mostly designed on rigid materials. Several researches have already been reported in the literature using rigid materials for different types of antenna sensors, including temperature sensing [16], crack sensing [17], strain sensing [18], and dielectric sensing [19]. Microstrip patch antennas are usually employed in sensing applications because they are characterized by several advantages: low manufacturing cost, low weight, durability and reliability, and small size. Patch antennas act as sensors through the interaction between dielectric properties and electromagnetic waves. For instance, a microstrip patch antenna-based sensor using flame retardant 4 (FR-4) substrate has been presented in [20]. The proposed antenna is employed as a sensor to detect different percentage of sugar and salt in terms of return loss based on the dielectric properties of the solution. In [21], a microstrip patch antenna sensor was printed on a Rogers (R03006) substrate. The presented antenna has been designed for temperature detection by subjecting a patch antenna bonded to various metal bases to thermal cycling. These types of substrates are not quite suitable for wearable antenna sensors as they cannot be stretched and bent. A wearable antenna sensor often should have additional characteristics such as robustness and flexibility, which claims the consideration of flexible nonconventional materials to exchange traditional printed circuit boards [22].

Wearable textile antenna sensors are becoming more and more essential in on-body applications in the last decade [23], due to their ability to detect microstructure deformations and human motions and to monitor and supervise the human health [24,25]. Compared with conventional antenna sensors, textile antenna sensors are able to be integrated on the outfits and they offer key features such as comfort, light weight, and washability. Various wearable flexible antenna sensors have been proposed in the literature. An example of a finger motion antenna sensor based on a dipole antenna proposed to realize dual function of sensing and communicating in the wireless sensor system was presented in [26]. Moreover, this dipole antenna sensor was attached on a glove to assess the human bending impact in the actual wearable device scenario. Taking into account the previous examples and as a future trend, the antenna sensor technology could be used in human machine interfacing, healthcare, robotics, and virtual reality.

Conference proceedings and full-text articles were chosen from a comprehensive search including diverse sources and databases such as ScienceDirect, Springer, Web of Science, and IEEE Xplore. Keywords were selected in each source as follows: (wearable OR flexible) AND (textile OR electro-textile) AND antenna sensor. The initial search returned 180 results. All results were screened and analyzed to eliminate duplicates and the final total was 83 studies, of which 33.4% were focused on the classification of antenna sensors, 52.3% were associated researches on technological feasibility and reliability, and the remaining 14.3% were investigative researches on scenario-based applications.

The Section 2 details the operating principle and the types of available antenna sensors in the literature. Section 3 presents a survey of the impact of certain characteristics of textile materials, different manufacturing techniques and particular examples and applications of flexible textile antenna sensors. Finally, Section 4 exhibits the main conclusions and future research directions and challenges related with antenna sensors.

## 2. Principle of Operation and Classification of Antenna Sensors

### 2.1. Principle of Operation

**Figure 1:**
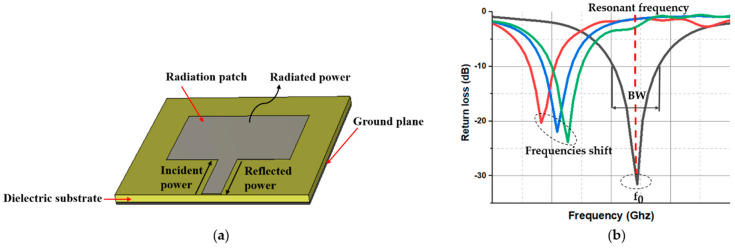
(**a**) Basic configuration of a microstrip antenna sensor and (**b**) illustration of frequencies shift of the antenna sensor.

In this section, a rectangular microstrip patch antenna is used to explain the principle of operation of an antenna sensor. A microstrip patch antenna contains four elements: a radiation patch, a dielectric substrate, a ground plane, and a transmission feed line as shown in Figure 1a. The radiation patch and the conductive ground plane (which can take any possible shape) are detached by a dielectric substrate. As a consequence, an effective electromagnetic resonance cavity allows radiation at particular frequencies [27]. The radiation patch is supplied by a microstrip feed line (Figure 1a), and an incident signal is provided. This signal is transmitted or reflected by the radiation patch. Consequently, the return loss of the microstrip patch antenna can be determined by the ratio between the reflected power and the incident power, also named “reflection coefficient” [21]. The radiation characteristics of the microstrip patch antenna can be characterized by the resonant bandwidth BW and the resonant frequency $f_{0}$. These two specifications can be extracted from the reflection coefficient (S11) [28]. The operating frequency of the antenna is determined as the frequency at which the reflection coefficient is minimum, i.e., little energy is reflected by the antenna and most of the incident power is radiated [29]. The resonant bandwidth of the antenna can be defined as the range of resonant frequencies at a given return loss, e.g., at −10 dB. In theory, all of these radiation parameters can be used to convert a physical quantity (strain, temperature, pressure, pH level, concentration of aqueous solution, etc.) into a measurable radiation parameter which leads to a resonance frequency shift (see Figure 1b).

### 2.2. Classification of Antenna Sensors

**Figure 2:**
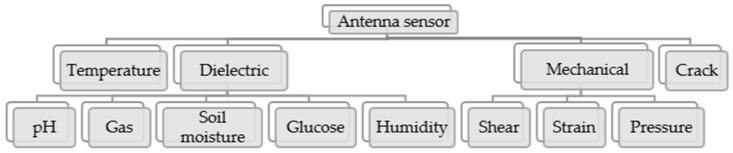
Different types of antenna sensors.

Antenna sensors can be classified into diverse categories as presented in the Figure 2. There are four main types of antenna sensors, namely, the temperature, dielectric, crack, and mechanical sensing. All these types of antenna sensors are able to detect changes using microwave signals or radio frequency (RF). Details of each types are described as follows.

#### 2.2.1. Dielectric Sensing

**Figure 3:**
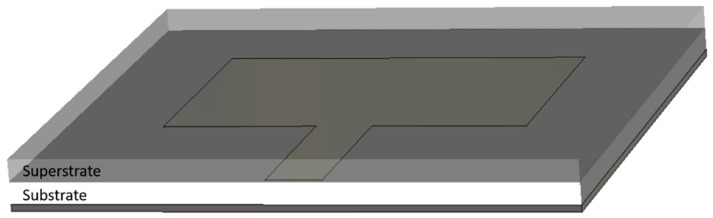
Configuration of an antenna dielectric sensor.

An antenna dielectric sensor can be represented by a patch antenna or by other standard planar antennas. Figure 3 presents a patch antenna where the radiation patch is covered with a dielectric material (superstrate). The choice of superstrate material depends on the selected measurand (humidity, salt and sugar, gas, etc.). The substrate can use different materials such as carbon nanotubes for gas sensing or polymer for humidity sensing or a textile material such as denim for blood glucose sensing [30]. However, the effective dielectric constant (relative permittivity) of the antenna sensor is provided by both the superstrate and the substrate [31]. Table 1 presents a summary of previously reported works on antenna dielectric sensors with different properties such as the measurand, the size, the operation frequency, material, and the sensing parameters.

#### 2.2.2. Strain Sensing

**Figure 4:**
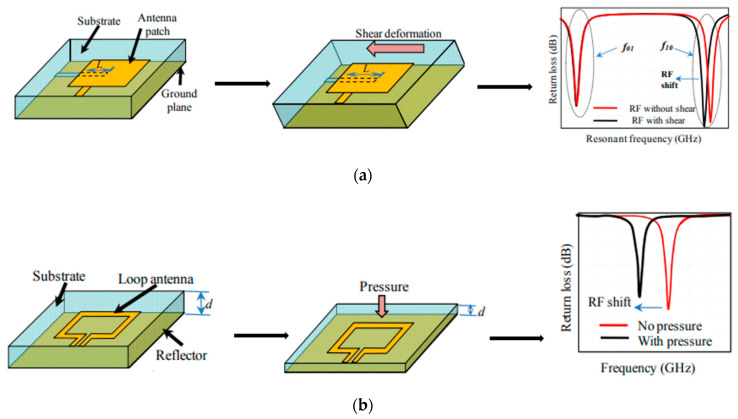
Antenna mechanical sensors for: (**a**) shear detection and (**b**) pressure detection, reproduced with permission from [45].

In order to check the structural integrity of the engineering components, strain is among the most important mechanical properties that must be used to quantify the deformation of a material [38]. Regarding types of strains, there are two strains: shear strain and normal strain. The first type is determined from the change of angle from an original value of 90° and the second type is related to the change in the size of a design compared to its original size [39]. Figure 4a shows the operating principle of an antenna sensor for shear detection. An antenna patch with a slot in the ground plane is used to visualize the effect of the shear on the behavior of the antenna. The principle of a loop antenna sensor for pressure detection is presented in Figure 4b. The variation of the parameter d in the geometry of the antenna structure detunes its operating frequency [40].

In order to provide accurate spatial resolution, it is recommended that the strain sensor presents a small size [41]. Different types of antenna mechanical sensors are summarized in Table 2.

#### 2.2.3. Temperature Sensing

The temperature of an antenna sensor is an important parameter to know as it indicates whether or not the antenna sensor is in control. This parameter is useful for many applications, e.g., food production, manufacturing process control, human health monitoring, etc. There are many different types of antenna temperature sensors available and all have different characteristics depending upon their application [46]. Table 3 presents some research works reported in the literature for temperature sensing with several properties: the type of the antenna sensor, the size, the operation frequency, the used materials, and the sensing parameters.

#### 2.2.4. Crack Sensing

**Figure 5:**
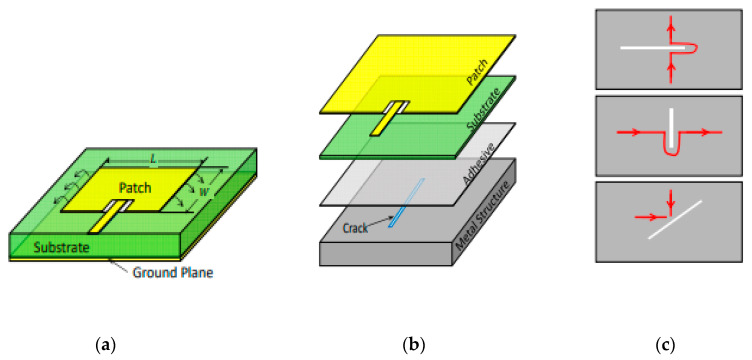
Antenna crack sensor: (**a**) schematic configuration of a patch antenna, (**b**) antenna sensor with crack, and (**c**) effect of cracks on the current model of the sensor [54].

In order to reduce catastrophic structural breakdowns, cracks must be monitored because they are a direct indicator of structural damage monitoring. In fact, it is important to know the length, direction, and location of the crack to gather sufficient information to maintain structural integrity [52]. Figure 5 presents a configuration of an antenna patch for crack detection. The direction and growth of the crack can be detected by observing the changes in the resonance frequency shifts. The resonance frequency shift of the crack antenna is generally much larger than the resonance frequency shift caused by strain or temperature [53]. Some examples for antenna crack sensors are given in Table 4.

## 3. Flexible Wearable Antenna Sensor

In order to provide good electrical performance as well as stability for the flexible devices, it is necessary to choose quality materials during manufacturing. The substrate selection for antenna sensor requires a low loss material so as to have better chances of increased antenna sensor efficiency when placed on the body. In fact, this is one of the important considerations for wearable electronics design [58]. Several flexible wearable antenna sensors are implemented on different types of materials such as papers [59], fabrics [60], and plastics [61]. Plastic substrates are neither recyclable nor biodegradable, as they affect environmental pollution and involve many health problems. Alternatively, textile materials are among the most internationally used and easily available materials for the design of flexible wearable antenna sensors with regard to body area networks (BANs).

### 3.1. Key Characteristics of Textile Materials in the Design of Antenna Sensors

The properties of the fabrics are determined from the properties of their constituent fibers and the structure of the fabric and/or the yarns. They are fibrous and porous materials, in which the pore size, fiber density, and air volume determine the general behavior, e.g., thermal insulation and air permeability [62]. Consistently, the density and thickness of fabrics can change with pressure as they are flexible, compressible, and stretchable materials. In addition, the fibers are constantly exchanging water molecules with the surrounding environment, which can sometimes affect their shape and properties [63]. It would be difficult to control these features in textile applications and, thus, it is necessary to know the influence of these factors on the behavior of the antenna sensor to reduce the undesirable and parasitic effects. Furthermore, the impact of the properties of textile materials on the performance of the antenna sensor is presented in this section.

#### 3.1.1. Relative Permittivity (Dielectric Constant) of the Fabrics

The dielectric permittivity of the substrate or material is one of the most important parameters affecting on the ability to transmit rapidly changing signals through the textile transmission line. The operation frequency and reflection coefficient in the transmission line can be affected by this phenomenon. The dielectric permittivity is defined as Equation (1):

(1) $$\varepsilon =\varepsilon_{0}\varepsilon_{r}=\varepsilon_{0}\left(\varepsilon_{r}^{\prime }-{j\varepsilon }_{r}^{\prime \prime }\right)$$

where $\varepsilon_{0}=$ 8.854 × 10−12 F/m is the permittivity of vacuum [64]. Generally, the moisture content, the temperature, the frequency, and also the surface roughness depend on the dielectric properties of the material under test [65]. The real part of the dielectric constant, $\varepsilon_{r}^{\prime }$, is also named “the relative permittivity”. It should be noted that this parameter is not constant in frequency. In addition, the material losses are typically given by the loss tangent, defined as $\tan\;\delta =\varepsilon_{r}^{\prime \prime }/\varepsilon_{r}^{\prime }$.

The dielectric properties of textiles are reviewed and studied in [66,67]. The textile materials dielectric behavior depends on the characteristics of the constituent polymers and fibers. Various experimental methods have been used to determine an accurate measurement of the dielectric characteristics of textiles. Among these techniques, there are the cavity perturbation method [68], the MoM-segment method [69], the free-space method [70], and the transmission line method [71]. Generally, textiles offer a very low relative permittivity (in comparison with typical rigid substrate materials for electronic applications) as they are very porous fabrics. Table 5 presents the dielectric properties of common commercial textile fabrics.

#### 3.1.2. Surface Resistivity of Fabrics

The electronic performance of fabrics can be determined by the surface resistance. Hence, the surface resistance is the ratio of a direct voltage applied to the current obtained from two electrodes placed on the surface of a material [73], and it also can be defined by the ratio between the DC voltage drop per unit length and the surface current per unit width. Surface resistivity is thus a property of the fabric considering a constant thickness, not depending on the design of the electrodes used for the measurement [62]. It is usually indicated by Ohm/square (Ω/sq).

#### 3.1.3. Regain of the Fabrics

Relative humidity (RH) of the fabrics is determined as the amount of water in a sample of air compared to the maximum amount of water the air can hold at a given temperature. It is expressed in a form of 0% to 100% [74]. In [65], some studies are presented on various textile fibers which indicate the relationship between relative humidity of the air and regain (amount of humidity present in a fabric calculated as a percentage of its oven-dry weight). Note that for the same relative humidity conditions, there are textile fibers with different humidity contents. For example, at 65% RH, cotton fiber could offer a regain of 7.5%, polyester fiber might offer a regain of 0.2%, and wool fiber might offer a regain of 14.5% [65]. Generally, the humidity absorption changes the properties of fibers, such as the effective permittivity or the mechanical rigidity. For this reason, fabric metrology is carried out at a specified temperature of 20 °C and relative humidity of 65% [62].

#### 3.1.4. Mechanical Deformations of the Fabrics

Textile fabrics are characterized by their good elasticity and flexibility, which makes them adaptive to curvature of the human body. However, after adapting to the topology of the surface, the structure is usually deformed and bent. These geometrical modifications influence the performance of the antenna sensor and they also lead to changes in the electromagnetic properties of textile fabrics [75]. In fact, the elongation and the bending of the dielectric fabric affect their thickness and their effective permittivity, which influences the resonance frequency of the antenna sensor. Furthermore, when the antenna sensor is compressed or elongated, the geometric accuracy decreases, affecting the behavior of the antenna sensor and, as a result, a resonant frequency shift can be produced.

### 3.2. Fabrication Methods for Wearable Antenna Sensor

Fabrication techniques are the determinants of the accuracy and manufacturing speed of low-cost wearable antenna sensor designs. The most popular wearable fabrication techniques are listed as follows: wet-etching [75], screen printing [76], inkjet printing [77], and embroidery methods [78]. To ensure durability, low cost, and high comfort to users in their daily wear, these techniques can be used for antenna sensors fabrication. An interesting review of these manufacturing methods is presented in [79,80,81]. Several of the aforementioned manufacturing techniques are discussed below.

#### 3.2.1. Screen Printing

To produce a lightweight and flexible antenna sensor, screen printing is a simple and economical approach used by many electronics manufacturers. In addition, the screen printing is an additive operation, which makes it environmentally friendly [82]. Instead of hiding the woven screen that has different thread densities and thicknesses, the mask with the required pattern is adjusted directly to the substrate where the conductive ink is handled and thermally annealed.

Moreover, the screen-printing technique faces diverse limitations. It comprises its limited number of realizable layers, lack of thickness control for the conductive layer, and low printing resolution. These aspects lead to the limited implementation of this technology, because wearable printing requires better precision for the convenient operation of the communications for wearable devices.

#### 3.2.2. Inkjet Printing

**Figure 6:**
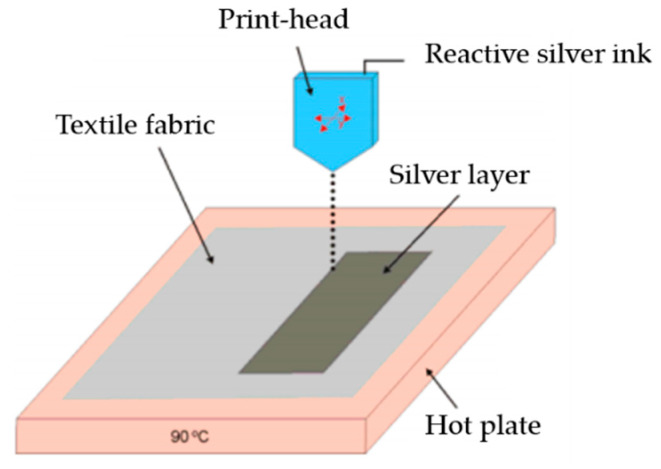
Silver deposition on textile surface using inkjet printing, reproduced with permission from [86].

Inkjet printing is one of the relatively low-cost printing technologies [83]. This technology is capable of producing a very high precision pattern due to its use of ink droplets of the size of up to a few picoliters [84]. Additionally, this technique allows the design pattern to be transmitted directly to the substrate with no requirement for masks. In addition, inkjet printing projects the single ink droplet from the nozzle to the required position, from which no waste is founded, which makes it among the economical manufacturing methods. This is a clear advantage in comparison with traditional etching technology, which has been generally used in industry [85]. The main drawbacks of inkjet printing technology are the incompatibility of certain types of conductive inks due to the larger particle size and clogging of the nozzles. Figure 6 presents an example of the inkjet-printing method using electroconductive layers on the surface of textile.

#### 3.2.3. Embroidery

**Figure 7:**
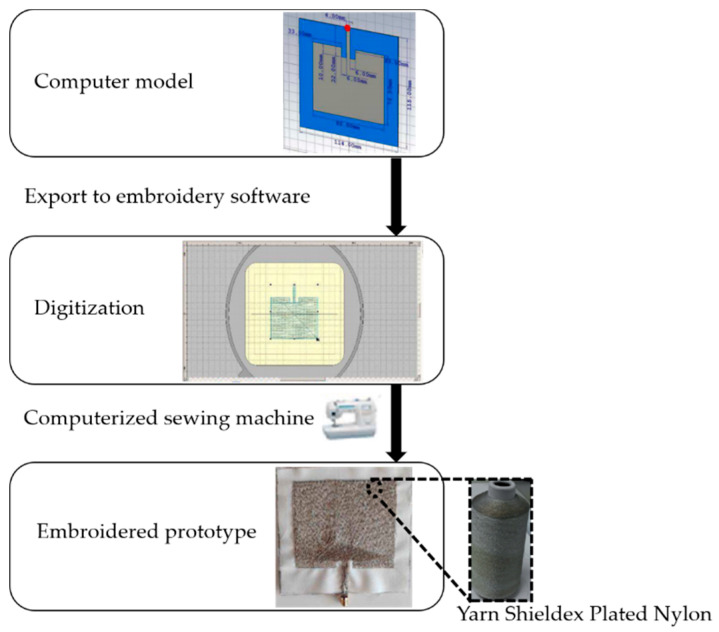
Embroidery process, reproduced with permission from [90].

This technique has been evolved to allow a digital image or layout to be directly embroidered using a computer-assisted embroidery machine. The embroidery manufacturing uses specialized conductive threads, from which the antenna sensor can be embroidered on the base substrate textile fabric. Before embroidering, it is very important to know the properties of the conductive threads that are going to be used (conductivity, DC resistance, and mechanical parameters), because when the conductive thread is characterized, it is then easier to find methods to improve the performance of the antenna sensor [87]. Therefore, the conductive threads must have adequate resistance and flexibility to avoid undesired breaks produced by high tensions in the embroidery machine [88]. Figure 7 depicts the embroidery technique, starting with the simulated design model to embroider the antenna integrated with the textile substrate.

Although embroidered antenna sensors are greatly considered as an ideal solution to replace traditional antennas in flexible electronics, compared with antenna sensors fabricate of metallic materials, they present some limitations, e.g., the embroidered geometry is much stretchable than metallic antenna sensors on inlays. This stretching impact, combined with the low resolution of the yarn stitches, makes fine geometries impractical [89]. The resistance of conductive yarn is much higher than metallic materials. They are either made of nylon cores coated with silver plating or carbon. The resistivity of the antenna sensors made from these yarns is order-of-magnitude higher than metallic ones even printed ones (made of silver paste or made of aluminum or copper).

#### 3.2.4. Comparison of Embroidery with Other Techniques

The embroidery process is advantageous over other techniques and embroidery machines are more recommended in the industry. This technique is easier to apply for mass production of clothing with integrated embroidered antenna sensors. In embroidery, the currents in the fabric flow along the yarns, making linear antenna sensors such as spirals or dipoles suitable for this fabrication technique, because it is very difficult to manufacture this type of structure using a Nora dell cloths or copper tape. With embroidery, the use of glue is not always a requirement to connect the textile layers together and also it allows to create reproducible geometries via computerized embroidery machines [91]. This can improve the washability of the clothing with the integrated antenna sensor.

### 3.3. Alternative Materials for the Deployment of Flexible Antenna Sensors

**Figure 8:**
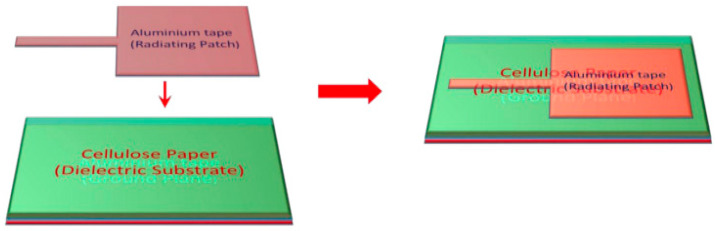
Manufacturing process of rectangular patch antenna sensor, reproduced with permission from [96].

Various flexible wearable antenna sensors are implemented on different types of materials such as Kapton polyimide [92], cellulose filter paper [93], polydimethylsiloxane (PDMS) film [94], and graphene film (FGF) [95]. These materials are being applied as promising candidates for innovative flexible antenna sensors. All of these antenna sensors are discussed in the next section. Furthermore, the graphene has attracted tremendous interest in wearable communication devices due to its outstanding electronic properties and performance. However, the applications of graphene in antenna sensors are limited because the use of single or few layer graphene films exhibit insufficient electrical conductivity and high sheet resistance. Figure 8 represents the manufacturing process of an antenna sensor. This antenna is fabricated on a cellulose paper substrate with a radiating patch of aluminum tape and a ground plane.

### 3.4. Applications and Specific Examples of Flexible Textile Antenna Sensor Designs

In current society, sensing applications are always based on some specific scenarios, which require different functions for the antenna sensors. Flexible antenna sensors are an attracted research orientation in the field, and there are some typical samples as listed in Table 6, including dipole antenna sensors [26], patch antenna sensors [93,95,96,97,98], and RFID tag sensors [99,100].

**Figure 9:**
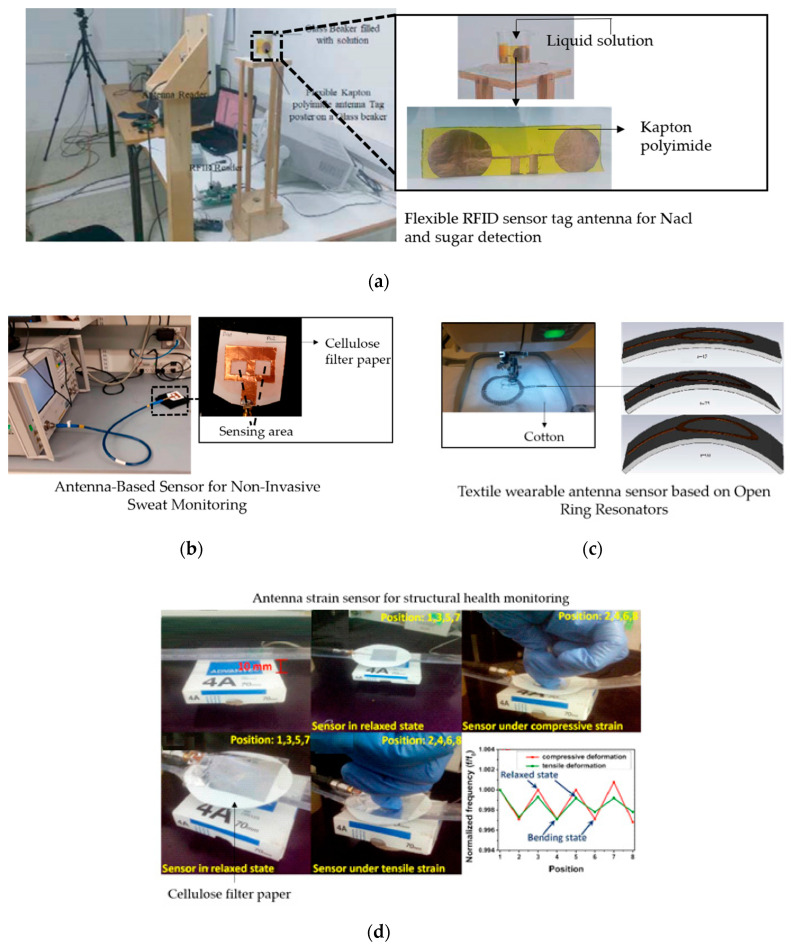
(**a**) Experimental setup for the measurements of the concentration of aqueous solutions, reproduced by courtesy of The Electromagnetics Academy [99]; (**b**) antenna sensor photo after absorbing saline solution [93]; (**c**) antenna sensor under bending stress, reproduced with permission from [101]; and (**d**) photographs of an antenna sensor in state tensile strain and compressive, reproduced with permission from [96].

A cellulose filter paper substrate for liquids detection was presented as shown in Figure 9b [93]. The patch antenna sensor utilized the special feature of the paper substrate, which is capable of absorbing liquids, such as sweat on the skin. The dielectric constant of the paper substrate is sensitive to the properties of the absorbed liquid through the 2 slots in the structure. In this paper, the feasibility of the sweat detection is validated by artificial sweat and salt solutions measurements, in which the frequency mainly shifted with changing NaCl (sodium chloride) concentration (8.5–200 mmol/L).

Compared with the mentioned flexible based antenna sensor for solution detection, another antenna sensor with RFID techniques [99] was designed to detect the concentration of NaCl (sodium chloride) solutions and sucrose solutions, which was printed on a polyimide substrate as shown in Figure 9a. The proposed RFID tag antenna was used to detect the solutions based on sensitivity to different levels of concentration. From tests with concentration levels from 0% to 80% at the different frequency points (864, 868, 915, and 926 MHz), the results revealed that sensitivity increases with the rise of concentration levels of NaCl and sucrose solutions.

For the body fluids detection field, another typical textile-based antenna sensor was proposed for the blood glucose monitoring as shown in Table 6 [97]. This proposed antenna sensor was designed to operate at 2.4 GHz in a noninvasive manner. In this work, the SAR analysis was taken into account since the antenna sensor would radiate towards the body when operating on the arm. In addition, the work revealed the SAR was also sensitive to the substrate thickness. For instance, when the thickness value was 4 mm, the SAR value would be 3.86 W/kg, which was lower than the safety limit value of 4 W/kg.

A microstrip patch antenna sensor was designed to detect temperature as detailed in Table 6 [98]. The proposed antenna sensor was embroidered on a cotton substrate and developed to operate at ISM band around 2.45 GHz. In this work, the relative permittivity of the substrate sensitive to the temperature was utilized to affect the resonant frequency points. Through this way, the low-cost solution for heat monitoring could be used for body temperature detection.

A textile-based antenna sensor was proposed for the research on bending impact as shown in Figure 9c [101]. This proposed antenna sensor was designed with an open ring resonator structure, which was sensitive to the bending levels of the structure. In this work, the antenna sensor was tested under different bending radius (45 and 90 mm) and results showed an output sensitivity from 0.4 to 2.2 MHz/mm. The feasibility and usefulness could be validated for developments of the antenna sensor based on textile materials with an open ring resonator structure.

A paper-based patch antenna sensor was proposed for strain detection as shown in Figure 9d [96]. The antenna sensor was fabricated by a radiation patch as the antenna and sensor, a layer of cellulose filter paper as the substrate, and an aluminum tape as the ground pane. In this work, after hundreds of bending cycles in the bending strain tests, the performance of the antenna sensor was still stable. In addition, through small cracks identification, bending angle analysis, and real human motion detection applied to gloves, the antenna sensor was fully evaluated and proved to be feasible to be used in medical, healthcare, and modern electronic device areas.

A graphene-based antenna sensor was proposed for high strain detection as listed in Table 6 [95]. This antenna sensor was designed to operate at 1.63 GHz with the flexible multilayer graphene film (FGF) whose conductivity reaches 106 S/m. In this work, the performance of the proposed antenna sensor with the special material was tested under tensile and compressive bending situations, respectively. In addition, compared with the antenna sensor with similar copper material, the strain sensitivity of the graphene-based antenna sensor was higher. The kind of antenna sensor had some good features in reversible deformability, mechanical flexibility, and structure stability, which made it suitable for some applications such as wireless strain sensing and wearable devices.

## 4. Conclusions and Future Research Directions

In the realization and implementation of wearable devices, flexible antenna sensors are getting more attention due to their conformal characteristics, lightweight, and low cost, being ideal for wireless communication and sensing applications. This review starts with the principle of operation and the types of antenna sensors used and their state-of-the-art including technologies to realize these devices. For any presented type, divers reference examples are provided. Next, a survey of the effect of some features of the wearable materials, including dielectrics and conductors, in the behavior of the antenna sensors is reported. The work also details some guidelines for the choice of materials for designing textile antenna sensors. The current advanced manufacturing techniques for flexible wearable antenna sensors are discussed. Finally, several applications and specific examples of flexible wearable antenna sensor designs are reviewed. Flexible antenna sensors are promising devices to enhance and boost the development of wireless communication technology and contribute to the miniaturization and improvement of performance of the future communication systems, especially with regard to wearable applications and human body area network scenarios.

Flexible wearable antenna sensors are a topic of increasing interest for industry and the scientific community. For this reason, some research key issues and challenges foreseen for future research in this area are presented as follows:

- Improving the precision and efficiency of the current manufacturing and measuring methods.
- Introducing new yarns and conductive fabrics in the market with less resistivity or higher conductivity.
- Introducing new flexible wearable materials for embroidery technique or new proposed manufacturing techniques.
- Introducing new antenna sensors based on textile substrates to operate on body.

Textile antenna sensors are expected to be used in various fields such as industry, healthcare, security, and so on. In addition, it is still a long way for wearable antenna sensors to improve their performance and increase their reliability in future applications. Further research is still needed to explore novel designs with new manufacturing processes and textile materials. New materials such as graphene and conductive ink on textile substrates have been employed in order to improve the performance of textile sensors and are promising candidates for next-generation antenna sensors.

Textile antenna sensors present great potential applications in many areas of life and production. In fact, the reported research in the scientific literature is mainly focused on the basic functions of textile antenna sensors such as humidity sensing, temperature sensing, and strain sensing. Nowadays, there are some uses for the textile antenna sensors that do not cover advanced functions and significant research is in progress. For example, there are wearable textile antenna sensors that can only detect the temperature without analyzing any other element such as bending and reliability, which requires more research. In addition, textile antenna sensors are more suitable for different applications such as for medical applications due to the various medical textiles employed for elderly or patients. Many types of parameters including moisture, strain, blood-glucose, and pH could be detected by means of textile antenna sensor systems. As a consequence, numerous scenarios could create many opportunities for new designs and applications of textile antenna sensors in the future.

## Figures and Tables

**Table 1:** Summary of previously reported works on antenna dielectric sensors.

Ref	Measurand	Type of Antenna	Size (mm^2^)	Freq	Material	Type of Material	Sensing Parameters
[11]	PH	Hexagonal split-ring resonator	19 × 23.35	3–20 GHz	^1^ PCB	FR-4	Transmission coefficient
[20]	Salt and sugar	Crescent-shaped patch	32 × 22	2.5–18 GHz	^1^ PCB	FR-4	Return loss
[32]	Humidity	H-shaped patch	90 × 85	880 MHz	Polymer	^2^ PEDOT: PSS/	Threshold power
[33]	Gas	Patch antenna	41 × 41	2.4 Ghz	^1^ PCB	Rogers RT/duroid 5880	Frequency shift
[34]	Soil moisture	Spiral antenna	12 × 12	2.48 GHz	^1^ PCB	FR-4	Frequency shift
[35]	Moisture content	Patch antenna	48 × 48	2.26 GHz	Polymer	^3^ PDMS	Frequency shift
[36]	Humidity	Patch antenna	30 × 20	38 GHz	Textile	Cotton	Frequency shift
[37]	Relative humidity	Split ring resonator	35 × 35	0–1.5 GHz	Polyimide	Kapton	Frequency shift

^1^ Printed circuit board. ^2^ Poly (3,4-ethylenedioxythiophene) polystyrene sulfonate. ^3^ Polydimethylsiloxane.

**Table 2:** List of antenna mechanical sensors.

Ref	Measurand	Type of Antenna	Size (mm^2^)	Freq	Material	Type of Material	Sensing Parameters
[39]	Strain	Dipole antenna	17 × 16	8–12 GHz	Polymer	Polyimide	Frequency shift
[42]	Pressure	Slot antenna	17.55 × 13.5	5.5 GHz	^1^ PCB	Rogers laminate RO4350b	Frequency shift
[43]	Strain	Patch antenna	94.58 × 52.36	1.8–2.4 GHz	Textile	Felt	Frequency shift
[44]	Shear and pressure	Patch antenna	12.1 × 66.9	6–7 Ghz	Polyimide	Kapton	Frequency shift

^1^ Printed circuit board.

**Table 3:** Summary of previously reported works on antenna temperature sensors.

Ref	Measurand	Type of Antenna	Size (mm^2^)	Freq	Material	Type of Material	Sensing Parameters
[47]	Body temperature	Patch antenna	10 × 6	38 GHz	Textile	Cotton	Frequency shift
[48]	Temperature	Slotted patch	38 × 38	900 MHz	^1^ PCB	FR-4	Frequency shift
[49]	Temperature	Rectangular patch	11.8 × 9.8	4.85 GHz 5.95 GHz	^1^ PCB	Rogers laminate RO3006	Frequency shift
[50]	Temperature	Patch antenna	13.6 × 10.9	2.4–2.8 GHz	^1^ PCB	Rogers RO3210	Frequency shift
[51]	Temperature	Patch antenna	71 × 64		Textile	Cotton	Frequency shift
						Jeans	
				2.45 GHz		Viscose	
						Lycra	
				9.5 GHz			
				38 GHz		Cotton	

^1^ Printed circuit board.

**Table 4:** Summary of previously reported works on antenna crack sensors.

Ref	Measurand	Type of Antenna	Size (mm^2^)	Freq	Material	Type of Material	Sensing Parameters
[53]	Crack orientation	Rectangular patch	15 × 12.75	5.75 GHz 6 GHz	^1^ PCB	Rogers laminate RO4350B	Frequency shift
[55]	Crack opening and growth	Rectangular patch	15 × 12.5	6.1 GHz 8.6 GHz	Polyimide	Kapton	Frequency shift
[56]	Crack	Patch antenna	35 × 20.6	2.4 GHz	^1^ PCB	Rogers RT/duroid 5880	Frequency shift
[57]	Crack and monitoring	Patch antenna	50.8 × 25.4	6.1 GHz 7.6 GHz	Polyimide	Kapton	Frequency shift

^1^ Printed circuit board.

**Table 5:** Dielectric properties of normal fabrics. Data from reference [72].

Nonconductive Fabric	$\varepsilon_{r}$	tan δ
${\mathrm{Cordura}}^{®}$	1.90	0.0098
Cotton	1.6	0.0400
100% polyester	1.90	0.0045
${\mathrm{Quartzel}}^{®}$ fabric	1.95	0.0004
Felt	1.215–1.225	0.016
Silk	1.75	0.012
Jeans	1.7	0.025
Fleece	1.17	0.0035
Denim	1.6–1.65	0.05

**Table 6:** List of some previously reported work of flexible textile antenna sensor.

Ref	Measurand	Type of Antenna	Size (mm^2^)	Freq	Material	Type of Material	Sensing Parameters
[26]	Finger postures	Dipole antenna	100 × 20	426 MHz	Filter paper	Cellulose filter paper	Frequency shift
[93]	Noninvasive Sweat	Patch antenna	50 × 60	2–4 GHz	Filter paper	Cellulose filter paper	Frequency shift
[95]	Strain	Patch antenna	35 × 27.4	1.63 GHz	Carbon	Graphene Film (FGF)	Frequency shift
[96]	Strain	Patch antenna	29.5 × 37.7	2.4 GHz	Filter paper	Cellulose filter paper	Frequency shift
[97]	Blood glucose	Patch antenna	40 × 40	2.4 GHz	Textile	Denim	Specific Absorption Rate
[98]	Temperature	Patch antenna	71 × 64	2.45 GHz	Textile	Cotton	Frequency shift
[99]	NaCl and sugar	RFID tag	100 × 32	860–960 MHz	Polyimide	Kapton	Frequency shift
[100]	Strain	RFID tag	100 × 20	866.6 MHz	Textile	Polyester	Frequency shift
